# Perceived Utility of Cognitive Behavioral Therapy in People With Bowel Disorders of Gut–Brain Interaction

**DOI:** 10.1111/nmo.70283

**Published:** 2026-03-08

**Authors:** Diego Izquierdo Veraza, Mohammed Rayyan Waseem, John Venezia, Anne Mary Montero, Sean Jones, Jhalka Kadariya, Anita Gupta, Huiping Xu, Andrea Shin

**Affiliations:** ^1^ Department of Medicine Indiana University School of Medicine Indianapolis Indiana USA; ^2^ Division of Gastroenterology and Hepatology, Department of Medicine Indiana University School of Medicine Indianapolis Indiana USA; ^3^ University of Alabama at Birmingham Birmingham Alabama USA; ^4^ Department of Biostatistics Indiana University School of Medicine Indianapolis Indiana USA; ^5^ Vatche and Tamar Manoukian Division of Digestive Diseases University of California Los Angeles California USA

**Keywords:** brain–gut axis, integrative care, psychological therapy, psychotherapy

## Abstract

**Background and Aims:**

Cognitive behavioral therapy (CBT) is an effective but underutilized treatment for bowel disorders of gut–brain interaction (DGBI). We aimed to examine attitudes and perceptions toward CBT in adults with and without bowel DGBI or other gastrointestinal (GI) diseases.

**Methods:**

We conducted an online survey on perceptions and experiences related to CBT. Presence of bowel DGBI was determined using Rome IV criteria. Responses were compared across individuals with bowel DGBI, other GI diseases, and controls, and between individuals with different types of bowel DGBI including irritable bowel syndrome (IBS), functional constipation (FC), and functional diarrhea (FDr). Associations between psychosocial factors and perceptions of CBT were examined.

**Results:**

Of 770 participants (268 with bowel DGBI), 70.2% reported CBT could be helpful. Barriers included lack of trained professionals, cost, and time or effort. Participants with bowel DGBI were more familiar with CBT (OR = 1.72, *p* < 0.001), but no more likely to have been offered CBT than controls. Those with other GI diseases had 4.3‐times higher odds of having been offered CBT. Attitudes toward providers recommending CBT and overall receptiveness to CBT did not differ among groups. Non‐White, non‐Black individuals were less likely to perceive CBT as helpful (OR = 0.61, *p* = 0.01), while Black participants were more willing to try CBT (OR = 1.73, *p* = 0.003). Participants with FDr were more likely to report CBT could be helpful than those with IBS (OR = 2.62, *p* = 0.035).

**Conclusions:**

Despite similar perceptions, patients with bowel DGBI are less frequently referred for CBT than those with other non‐DGBI GI diseases. Sociocultural differences may also influence beliefs. Strategies for access expansion, early referrals, and culturally competent care will be essential for effectively integrating CBT into bowel DGBI management.

## Key Points

- It is not known if other factors beyond access‐related limitations hinder utilization of cognitive behavioral therapy (CBT) for patients with bowel disorders of gut–brain interaction (DGBI).
- Many adults with bowel DGBI perceive CBT as potentially beneficial, yet barriers including apprehension, underutilization by providers, and limited availability of time and resources may preclude its use. Sociocultural factors may also influence patients' perspective of CBT.
- Strategies to expand access, promote earlier referrals, and enhance culturally competent care represent potential pathways for increasing integration of CBT into the management of bowel DGBI.

## Graphical abstract

In an online survey, 70% of adults view cognitive behavioral therapy (CBT) as helpful. Cost, access, and time constraints are barriers. Those with bowel disorders of gut–brain interaction (DGBI) are familiar with CBT, but only those with non‐DGBI gastrointestinal diseases are more frequently offered CBT than controls.

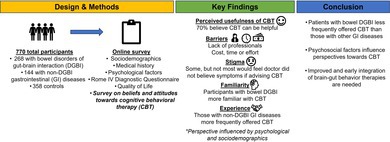

**Abbreviations**
ACE: adverse childhood experiences
CBT: cognitive behavioral therapy
DGBI: disorders of gut–brain interaction
FC: functional constipation
FDr: functional diarrhea
GI: gastrointestinal
HADS: hospital anxiety and depression scale
IBS: irritable bowel syndrome
IQR: interquartile rage
mTURK: mechanical Turk
OR: odds ratio
PTSD: post‐traumatic stress disorder
QOL: quality of life
SD: standard deviation
SF‐12: 12‐item short form survey

## 1 Introduction

Irritable bowel syndrome (IBS), functional diarrhea (FDr), and functional constipation (FC) are common bowel disorders that belong to a larger group of disorders known as disorders of gut‐brain interaction (DGBI) [1]. Estimated global prevalence rates of IBS, FDr, and FC range from 4%–10%, 9%–12%, and 4%–5%, respectively, depending on the diagnostic criteria used [2]. Bowel DGBI are associated with reduced health‐related quality of life (QoL), increased socioeconomic burden, and higher psychological distress [3]. Dysregulation of the gut‐brain axis and biopsychosocial factors, including prior abuse, anxiety, and depression, may influence the development, severity, and frequency of symptoms [4, 5, 6]. Identifying optimal treatments can be challenging, and standard therapies including lifestyle modifications or pharmacotherapies may be insufficient for some [7]. Developing individualized treatment plans that target modifiable aspects of the disease experience, including cognitive and emotional factors through multi‐faceted approaches, could enhance responses to treatment and improve patient outcomes [8].

Given the crucial role of centrally mediated mechanisms and psychological comorbidities in DGBI, brain–gut behavior therapies (BGBT) may be offered as part of an integrated care model to augment medical treatment, reduce symptoms, and improve overall well‐being [9, 10]. Data from clinical trials have generated substantial evidence supporting the efficacy of various forms of BGBT including cognitive behavioral therapy (CBT) [11, 12, 13]. Different types of BGBT rely on various techniques and target specific cognitive, behavioral, and emotional facets that contribute to gut‐brain dysregulation. For example, CBT may target psychological stress, negative emotion, maladaptive cognitive processes or behaviors, fear avoidance or fear of gastrointestinal (GI) symptoms, somatization, early life adversity, or psychological comorbidity and somatization while other techniques such as gut‐directed hypnotherapy may focus primarily on visceral hypersensitivity, maladaptive cognitions, and somatization [9, 10]. CBT is one of the most well‐studied BGBT that targets maladaptive behaviors and coping skills that have developed in response to symptoms and/or stress [10]. Studies have demonstrated that CBT is associated with improved symptoms and QoL and may even be as effective as medication [14] and that baseline traits may predict therapeutic success [15]. In a recent systematic review and network meta‐analysis, only group CBT was more efficacious than a control intervention for patients with global IBS symptoms that were refractory to treatment [16]. Despite the robust evidence supporting the role of CBT, gaps [17] between evidence‐based recommendations and implementation in clinical practice persist [9]. Reasons for limited integration of CBT into routine management of patients with bowel DGBI are not entirely clear. Lack of access to trained providers is a primary barrier. However, other factors including fear of stigmatization or skepticism may contribute. In some cases, patients may feel dismissed by healthcare providers or that their symptoms are being inappropriately labeled as psychosomatic in nature [18]. Relatively little research has been conducted to examine the issues that impact participation in CBT from the patient perspective. To identify potential barriers that have not yet been recognized but could be addressed, it is necessary to evaluate patient perceptions on the role of CBT including patient‐reported attitudes, beliefs, and experiences with CBT and whether these perceptions differ across patients with and without digestive diseases and/or symptoms. Therefore, we aimed to evaluate perspectives on CBT among the general public including individuals with and without lower bowel DGBI.

## 2 Materials and Methods

### 2.1 Study Design and Participants

We conducted a cross‐sectional online survey among US community‐based adults ≥ 18 years examining health‐related QoL, bowel symptoms, sociodemographics, psychological factors, and opinions on CBT from July to November of 2020. The study was approved by the Indiana University Institutional Review Board. Without prior knowledge of their current health status, individuals who are enrolled in Amazon's mechanical Turk (mTurk), a crowdsourcing website used for the completion of requester‐directed tasks, were invited to participate with web‐based survey invitations. Recruits from mTurk have been shown to approximate the demographics of American adult internet users [19]. Individuals with > 95% approval on mTurk and a US‐based IP address were eligible for participation.

### 2.2 Study Procedures

Survey invitations with a general description of the study and instructions for accessing the survey were distributed through the secure web‐based platform. Consent for the survey participation was implied (no written signature required). Data on sociodemographic variables, presence of bowel DGBI, history of other non‐DGBI gastrointestinal (GI) diseases, health‐related QoL, psychological factors (anxiety, depression, possible post‐traumatic stress disorder [PTSD]), environmental factors (e.g., childhood adversity), and beliefs and attitudes toward CBT (Supporting Information) were collected using a series of questionnaires including the Rome IV Diagnostic [20] questionnaire for bowel disorders, 12‐Item Short Form Survey (SF‐12) [21], Hospital Anxiety and Depression Scale (HADS) [22], PTSD Checklist (PCL‐5) [23], and Adverse Childhood Experiences (ACE) [24] questionnaire. Prior to answering questions pertaining to CBT, participants were given a general description of CBT, and questions were framed in the context of considering the role of CBT for any physical symptom, emotional health, or well‐being rather than CBT for GI‐specific symptoms. An attention check question was embedded into the survey to ensure respondents were engaged.

### 2.3 Study Endpoints

The primary outcome was perceived usefulness of CBT (belief that CBT could be helpful). Secondary outcomes included perceived barriers to CBT, attitudes toward providers recommending CBT, willingness to try CBT, perceived stigma of participating in CBT, and CBT familiarity. Outcomes were compared across adults with no bowel DGBI or GI diseases (i.e., controls), bowel DGBI, and other non‐DGBI GI diseases (e.g., inflammatory bowel disease, celiac disease, microscopic colitis, or colorectal cancer). Among those with bowel DGBI, responses were compared across DGBI subtypes. Additional analyses were conducted to examine associations between psychological factors, socioeconomic status, education, and childhood adversity with receptiveness to CBT.

### 2.4 Statistical Considerations

Data were summarized using the mean and standard deviation (SD), median and interquartile range (IQR), and frequencies and proportions. Comparisons were performed using the Kruskal‐Wallis and Pearson chi‐squared tests for continuous and categorical variables. Multivariable analyses were conducted using binary logistic regression models controlling for age, sex, and sociodemographics. Additional multivariable analyses examining associations of psychological variables, sociocultural factors, and clinical group with perceptions of CBT and willingness to try CBT were performed using ordered logistic regression with all covariates plus psychological comorbidities. Variable selection was conducted using the lasso approach where the tuning parameter is selected to minimize the 5‐fold cross validation error.

## 3 Results

### 3.1 Participant Characteristics

**FIGURE 1:**
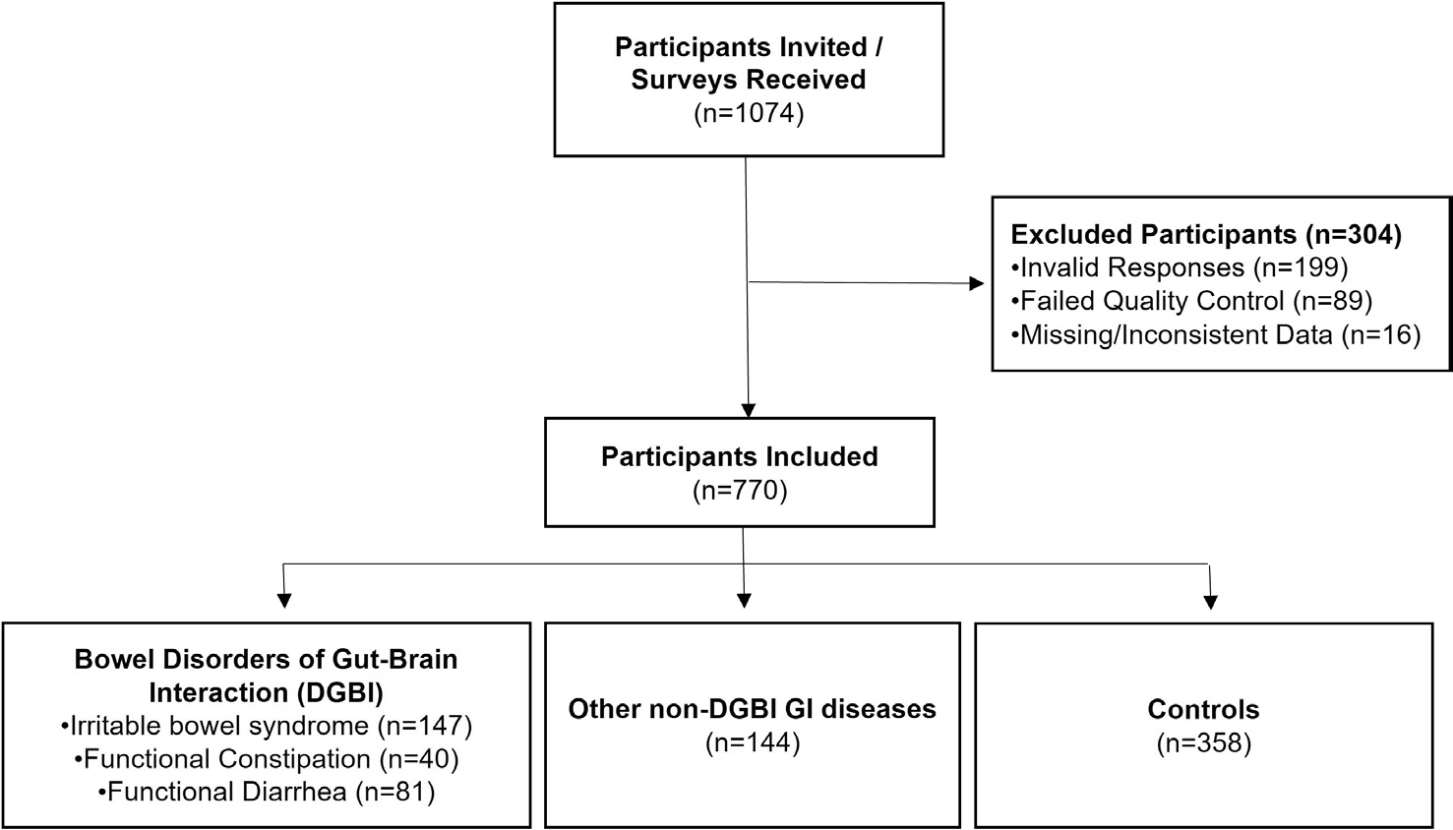
Study flow diagram.

The final cohort included 770 individuals (40.1% female, 68.2% Caucasian, mean age [SD] = 36.8 years [±11.1], *n* = 268 with bowel DGBI, *n* = 144 with other non‐DGBI GI diseases, *n* = 358 controls) after excluding participants with missing or inconsistent data and those who did not pass the quality control check (Figure 1). Age, gender, household income, and education levels did not differ across groups (Table 1). The number of Hispanic participants and rates of smoking and alcohol intake were higher in the bowel DGBI group. There were significant differences in SF‐12, HADS, ACE, and PCL‐5 scores (Table 2) across groups. Among 268 participants (Table 3) who satisfied Rome IV criteria for bowel DGBI (*n* = 147 IBS, *n* = 40 FC, *n* = 81 FDr), mean age (±SD) was 36.7 (±11.1) years; *n* = 107 (39.9%) were women and *n* = 179 (66.8%) were Caucasian. Smoking or tobacco use was more prevalent in IBS (*p* = 0.028). There were no significant differences in age, race, gender, marital status, alcohol intake, or education across patients with different bowel DGBI types. HADS anxiety scores, PCL‐5, and the physical component of SF‐12 scores were poorest in IBS (Table 4).

**TABLE 1:** Baseline characteristics of study participants.

	Bowel DGBI (*N* = 268)	Other GI disease (*N* = 144)	Controls (*N* = 358)	*p*
Age in years, median (IQR)	33 (28–43)	32 (28–41)	35 (29–45)	ns
Women	107 (39.9%)	57 (39.6%)	145 (40.5%)	ns
Race (*n* = 4 missing)				0.05
Caucasian	179 (67.0%)	93 (64.6%)	253 (71.3%)	
African American	52 (19.5%)	20 (13.9%)	60 (16.9%)	
Other	36 (13.5%)	31 (21.5%)	42 (11.8%)	
Hispanic	100 (38.6%)	48 (33.8%)	63 (18.1%)	< 0.001
Marital status				0.07
Married or long‐term partner	228 (85.1%)	110 (76.4%)	285 (79.6%)	
Single, widowed, or divorced	40 (14.9%)	34 (23.6%)	73 (20.4%)	
Education				ns
High school or technical/vocational degree	20 (7.5%)	12 (8.3%)	34 (9.5%)	
Bachelor's degree	168 (62.7%)	90 (62.5%)	243 (67.9%)	
Master's or doctorate degree	80 (29.9%)	42 (29.2%)	81 (22.6%)	
Doctorate degree	2 (0.7%)	3 (2.1%)	3 (0.8%)	
Technical degree or vocational	7 (2.6%)	3 (2.1%)	3 (0.8%)	
Current smoker or tobacco user	178 (66.4%)	91 (63.2%)	149 (41.6%)	< 0.001
Alcohol consumption				< 0.001
Never	36 (13.4%)	27 (18.8%)	81 (22.6%)	
Rarely	92 (34.3%)	48 (33.3%)	126 (35.2%)	
Occasionally	78 (29.1%)	37 (25.7%)	115 (32.1%)	
Regularly or frequently	62 (23.1%)	32 (22.2%)	36 (10.1%)	
Annual household income				ns
Less than $49,999	91 (34.0%)	58 (40.3%)	134 (37.4%)	
$50,000 to $74,999	101 (37.7%)	53 (36.8%)	124 (34.6%)	
$75,000 or more	76 (28.4%)	33 (22.9%)	100 (27.9%)	

*Note:* Median and interquartile range (IQR) of age are reported due to the skewness of the age distribution, with *p*‐value calculated based on the Kruskal–Wallis test. *p* values for categorical variables are based on the Pearson's chi‐squared test.

Abbreviations: DGBI, disorders of gut–brain interaction; GI, gastrointestinal.

**TABLE 2:** Comparisons of psychological factors across all groups (all *p* < 0.001).

	Bowel DGBI (*N* = 268)	Other GI disease (*N* = 144)	Controls (*N* = 358)
SF‐12, median (IQR)			
Physical component score	36 (33–41)	37 (34–40)	42 (37–52)
Mental component score	42 (37–46)	40 (37–45)	44 (39–50)
HADS anxiety score, median (IQR)	12 (10–14)	12 (9–13)	10 (6–12)
HADS anxiety			
Normal (0–7)	35 (13.1%)	20 (13.9%)	114 (31.8%)
Borderline abnormal (8–10)	43 (16.0%)	39 (27.1%)	85 (23.7%)
Abnormal (11–21)	190 (70.9%)	85 (59.0%)	159 (44.4%)
HADS depression score, median (IQR)	8 (7–10)	9 (7–11)	7 (4–10)
HADS depression			
Normal (0–7)	103 (38.4%)	43 (29.9%)	184 (51.4%)
Borderline abnormal (8–10)	108 (40.3%)	63 (43.8%)	113 (31.6%)
Abnormal (11–21)	57 (21.3%)	38 (26.4%)	61 (17.0%)
ACE score, median (IQR)	4 (3–6)	4 (3–6)	3 (1–4)
Presence of early adverse life events	253 (94.4%)	132 (91.7%)	302 (84.4%)
PCL‐5 total score, median (IQR)	46 (39–56)	41 (32–55)	38 (15–47)
Provisional PTSD diagnosis	222 (82.8%)	111 (77.1%)	218 (60.9%)

*Note:* *p*‐values are calculated based on the Kruskal–Wallis test and Pearson's chi‐squared test.

Abbreviations: ACE, adverse childhood experiences; HADS, Hospital Anxiety and Depression Scale; IQR, interquartile range; PCL‐5, PTSD Checklist; SF‐12, 12‐Item Short Form Survey.

**TABLE 3:** Baseline characteristics among participants with irritable bowel syndrome (IBS), functional constipation (FC), and functional diarrhea (FDr).

	IBS (*N* = 147)	FC (*N* = 40)	FDr (*N* = 81)	*p*
Age in years, median (IQR)	35.9 (11.3)	36.2 (10.9)	38.1 (10.8)	ns
Women	54 (36.7%)	18 (45.0%)	35 (43.2%)	ns
Race				ns
Caucasian	102 (69.4%)	30 (75.0%)	47 (58.8%)	
African American	26 (17.7%)	8 (20.0%)	18 (22.5%)	
Other	19 (12.9%)	2 (5.0%)	15 (18.8%)	
Hispanic (*n* = missing)	56 (38.6%)	11 (28.9%)	33 (43.4%)	ns
Marital status				ns
Married or long‐term partner	127 (86.4%)	32 (80.0%)	69 (85.2%)	
Single or divorced	20 (13.6%)	8 (20.0%)	12 (14.8%)	
Education				ns
High school or technical/vocational degree	11 (7.5%)	3 (7.5%)	6 (7.4%)	
Bachelor's degree	91 (61.9%)	31 (77.5%)	46 (56.8%)	
Master's or doctorate degree	45 (30.6%)	6 (15.0%)	29 (35.8%)	
Current smoker or tobacco user	106 (72.1%)	20 (50.0%)	52 (64.2%)	0.03
Alcohol consumption				ns
Never	19 (12.9%)	5 (12.5%)	12 (14.8%)	
Rarely	46 (31.3%)	17 (42.5%)	29 (35.8%)	
Occasionally	41 (27.9%)	13 (32.5%)	24 (29.6%)	
Regularly or frequently	41 (27.9%)	5 (12.5%)	16 (19.8%)	
Annual household income				ns
Less than $49,999	48 (32.7%)	14 (35.0%)	29 (35.8%)	
$50,000 to $74,999	55 (37.4%)	16 (40.0%)	30 (37.0%)	
$75,000 or more	44 (29.9%)	10 (25.0%)	22 (27.2%)	

*Note:* Median and IQR of age are reported due to the skewness of the age distribution, with *p* value calculated based on the Kruskal–Wallis test. *p* values for categorical variables are calculated based on the Pearson chi‐squared test.

**TABLE 4:** Comparisons of psychological factors among adults with bowel disorders of gut–brain interaction (DGBI).

	IBS (*N* = 147)	FC (*N* = 40)	FDr (*N* = 81)	*p*
SF‐12, median (IQR)				
Physical component score	34 (31–38)	39 (36–46)	38 (34–41)	< 0.001
Mental component score	42 (37–46)	44 (38–49)	41 (37–46)	0.48
HADS anxiety score, median (IQR)	13 (11–14)	10 (7–12)	12 (10–14)	< 0.001
HADS anxiety				< 0.001
Normal (0–7)	10 (6.8%)	13 (32.5%)	12 (14.8%)	
Borderline abnormal (8–10)	23 (15.6%)	8 (20.0%)	12 (14.8%)	
Abnormal (11–21)	114 (77.6%)	19 (47.5%)	57 (70.4%)	
HADS depression score, median (IQR)	9 (7–10)	8 (5–9)	8 (6–10)	0.28
HADS depression				0.6
Normal (0–7)	51 (34.7%)	18 (45.0%)	34 (42.0%)	
Borderline abnormal (8–10)	63 (42.9%)	16 (40.0%)	29 (35.8%)	
Abnormal (11–21)	33 (22.4%)	6 (15.0%)	18 (22.2%)	
ACE total score, median (IQR)	4 (3–7)	4 (3–5)	4 (3–7)	0.07
Presence of early adverse life (EAL) events	140 (95.2%)	36 (90.0%)	77 (95.1%)	0.42
PCL‐5 total score, median (IQR)	50 (42–59)	39 (31–44)	44 (38–50)	< 0.001
Provisional PTSD diagnosis	131 (89.1%)	30 (75.0%)	61 (75.3%)	0.011

*Note:* *p* values are calculated based on Kruskal–Wallis test for continuous variables and Pearson's chi‐squared test for categorical variables.

Abbreviations: ACE, adverse childhood experiences; FC, functional constipation; FDr, functional diarrhea; HADS, Hospital Anxiety and Depression Scale; IBS, irritable bowel syndrome; PCL‐5, PTSD Checklist; SF‐12, 12‐Item Short Form Survey.

In the collective cohort, 70.2% reported that CBT could be helpful for many conditions. Most believed there was some risk (93%). CBT risk was commonly compared to medications (45.7%) and risks encountered in everyday life (32.5%). More than a quarter also compared risks of CBT to risks of surgery (26.9%). Many identified a lack of available professionals (38%) and cost (42%) as barriers. Approximately 25% cited effort and the time‐consuming nature of CBT as barriers. Only 12% had ever been offered CBT. Of those who knew others who tried CBT, 90% reported benefit. Many (39%) reported they would feel their doctor did not believe their symptoms if CBT were advised, but 62% would still try it and 84% felt participating would not negatively impact how others viewed them.

### 3.2 Perceptions of CBT in Adults With Bowel DGBI, GI Diseases, and Controls

**FIGURE 2:**
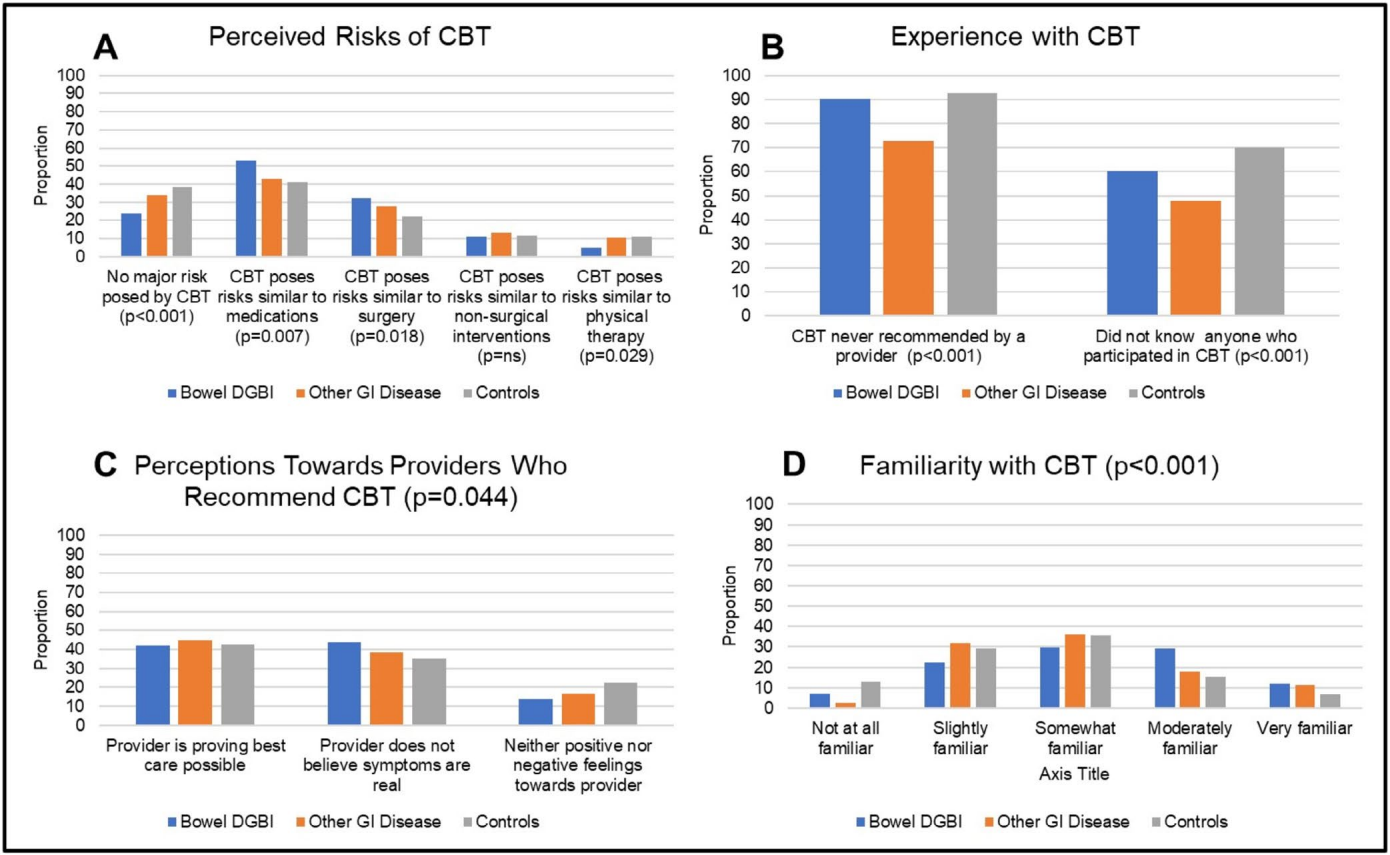
Perceptions and beliefs regarding cognitive behavioral therapy (CBT) among adults with bowel disorders of gut–brain interaction (DGBI), other gastrointestinal (GI) diseases, and controls. Panel A = proportion within each group who indicated that response for perceived risks. Panel B = response patterns for experiences with CBT. Panel C = response patterns for perceptions of providers who recommend CBT (overall *p* < 0.05). Panel D = response patterns for familiarity with CBT (overall *p* < 0.001).

In unadjusted analyses, overall receptiveness to CBT (belief that CBT could be helpful for any condition) did not differ across groups (*p* = ns). Among those who indicated CBT as sometimes or rarely helpful, responses to questions on what conditions would benefit from CBT or perceived barriers to CBT did not differ across groups (both *p* = ns). Significant differences were observed across groups in the degree of perceived risk posed by CBT (Figure 2), experiences with CBT (both *p* < 0.001), perceptions of providers who recommended CBT (*p* = 0.044), and familiarity with CBT (*p* < 0.001). Differences included a greater proportion of controls comparing CBT risks to everyday life and a greater proportion of those with bowel DGBI comparing risks of CBT to medications and surgery. Of those with bowel DGBI, 44% reported that if their provider recommended CBT, they would perceive their provider as implying that their symptoms were not real compared to 38.5% of participants with other non‐DGBI GI diseases and 35% of controls. Those with other non‐DGBI GI diseases more frequently had CBT recommended to them or knew others who tried CBT (both *p* < 0.001).

In multivariable analysis (Table 5), pairwise comparisons demonstrated that overall receptiveness to CBT, perceived barriers to CBT, perceptions of providers who recommended CBT, willingness to pursue CBT if recommended, and beliefs on how they would be viewed by others after participating in CBT did not differ between groups. Those with bowel DGBI were less likely to report that CBT posed no major risk (OR = 0.668, *p* = 0.047) or compared risks of CBT to physical therapy (OR = 0.463, *p* = 0.025) and more likely to compare risks of CBT to surgery (OR = 1.52, *p* = 0.037), prefer that CBT to be explained through a one‐time visit with a therapist (OR = 1.691, *p* = 0.03), and report a higher degree of familiarity with CBT (OR = 1.719, *p* < 0.001) than controls. Those with other non‐DGBI GI diseases had 4.3 times higher odds of having been offered CBT and 2.4 times higher odds of knowing others who tried CBT (both *p* < 0.001) than controls. The odds of having been offered CBT did not differ between those with bowel DGBI or controls (*p* = ns).

**TABLE 5:** Multivariable comparisons on perceptions of cognitive behavioral therapy (CBT) among adults with and without bowel disorders of gut–brain interaction (DGBI).

Data show odds ratios (95% CI)	Bowel DGBI (*N* = 268)	Other GI disease (*N* = 144)	Controls (*N* = 358)
Belief that CBT can be helpful	1.068 (95% CI: 0.783–1.457, *p* = ns)	1.380 (95% CI: 0.951–2.004, *p* = ns)	Reference
Reasons preventing CBT^a^			
Too much work and effort	1.003 (95% CI: 0.677–1.487, *p* = ns)	0.982 (95% CI: 0.613–1.574, *p* = ns)	Reference
Too costly	1.098 (95% CI: 0.772–1.560, *p* = ns)	0.858 (95% CI: 0.561–1.312)	reference
No available professionals	1.176 (95% CI: 0.824–1.679, *p* = ns)	1.184 (95% CI: 0.774–1.810, *p* = ns)	Reference
Too time‐consuming	1.021 (95% CI: 0.688–1.515, *p* = ns)	1.052 (95% CI: 0.659–1.679, *p* = ns)	Reference
No obvious reasons	0.754 (95% CI: 0.421–1.352, *p* = ns)	0.868 (95% CI: 0.436–1.727, *p* = ns)	Reference
Same risks as CBT^a^ ^s^			
No major risk beyond everyday life	0.668 (95% CI: 0.448–0.994, *p* = 0.047)	1.041 (95% CI: 0.659–1.645, *p* = ns)	Reference
Medications	1.273 (95% CI: 0.893–1.817, *p* = ns)	0.831 (95% CI: 0.541–1.277, *p* = ns)	Reference
Surgeries	1.520 (95% CI: 1.026–2.252, *p* = 0.037)	1.252 (95% CI: 0.777–2.016, *p* = ns)	Reference
Nonsurgical interventions	0.817 (95% CI: 0.474–1.408, *p* = ns)	0.956 (95% CI: 0.510–1.792, *p* = ns)	Reference
Physical therapy	0.463 (95% CI: 0.236–0.910, *p* = 0.025)	0.974 (95% CI: 0.500–1.900, *p* = ns)	Reference
Provider ever recommended CBT (*n* = 2 missing)	1.250 (95% CI: 0.695–2.246, *p* = ns)	4.321 (95% CI: 2.463–7.581, *p* < 0.001)	reference
Know someone who tried CBT? (*n* = 2 missing)	1.306 (95% CI: 0.907–1.882, *p* = ns)	2.361 (95% CI: 1.543–3.612, *p* < 0.001)	Reference
Perception of provider doctor if they recommend CBT (*n* = 8 missing)			
Providing the best care possible	1.442 (95% CI: 0.883–2.357, *p* = ns)	1.411 (95% CI: 0.799–2.490, *p* = ns)	Reference
Does not believe my symptoms are real	1.590 (95% CI: 0.965–2.620, *p* = ns)	1.151 (05% CI: 0.638–2.076, *p* = ns)	Reference
Likelihood of trying CBT if recommended by provider	1.262 (95% CI: 0.924–1.724, *p* = ns)	1.230 (95% CI: 0.849–1.782, *p* = ns)	Reference
Participating in CBT would result in others to view you positively	0.754 (95% CI: 0.548–1.036, *p* = 0.082)	0.862 (95% CI: 0.591–1.259, *p* = ns)	Reference
How would you like CBT explained?			
One‐time visit with therapist	1.691 (95% CI: 1.051–2.720, *p* = 0.03)	1.299 (95% CI: 0.748–2.257, *p* = ns)	Reference
Written information	1.441 (95% CI: 0.875–2.372, *p* = ns)	1.260 (95% CI: 0.708–2.242, *p* = ns)	Reference
On my own	1.592 (95% CI: 0.925–2.739, *p* = 0.093)	0.994 (95% CI: 0.512–1.929, *p* = ns)	Reference
Greater familiar with CBT prior to this study	1.719 (95% CI: 1.264–2.337, *p* < 0.001)	1.378 (95% CI: 0.956–1.985, *p* = 0.085)	Reference

^a^ Participants could select more than one response.

### 3.3 Psychological and Sociocultural Factors

In multivariable analyses with inclusion of psychosocial variables, race, mental component score, and depression levels were significantly associated with perceived benefit of CBT and willingness to try CBT. Non‐White and non‐Black participants were less likely (OR = 0.612, *p* = 0.012) to perceive CBT as helpful than White participants, while Black participants were more willing to try CBT (OR = 1.732, *p* = 0.003) than White participants. Participants with borderline (OR = −0.572, *p* ≤ 0.001) and abnormal (OR = 0.415, *p* ≤ 0.001) levels of depression and those with higher mental component scores (OR = 0.970, *p* = 0.003) were less likely to perceive CBT as helpful. Those with borderline (OR = 0.466, *p* < 0.001) or abnormal (OR = 0.431, *p* < 0.001) levels of depression, provisional PTSD (OR = 0.621, *p* = 0.008), and higher mental component scores (OR = 0.975, *p* = 0.008) were less willing to try CBT. After inclusion of psychological variables, participants with other non‐DGBI GI diseases were more likely to find CBT helpful (OR = 1.52, *p* = 0.025) while perceived benefit with CBT did not differ between those with bowel DGBI and controls (*p* = ns). In contrast, participants with bowel DGBI were more willing to try CBT than controls (OR = 1.732, *p* = 0.003).

### 3.4 Perceptions of CBT by Bowel DGBI Type

Of those with bowel DGBI, 44.6% indicated CBT was sometimes or probably helpful for many conditions and 27% indicated CBT was helpful for almost any condition. After covariate adjustment, participants with FDr were more likely to perceive CBT as helpful than those with IBS (OR = 2.17, *p* = 0.005) and know someone who tried CBT than those with FC (OR = 2.62, *p* = 0.035). Those with IBS (OR = 2.18, *p* = 0.017) or FC (OR = 2.68, *p* = 0.026) were more likely to report lack of professionals as a barrier than those with FDr. Those with FC (OR = 0.28, *p* < 0.01) were less familiar with CBT than those with IBS. Inclusion of psychosocial variables in multivariable models did not change overall associations of perceived helpfulness of CBT with bowel DGBI group.

## 4 Discussion

In this study, 70% of adults viewed CBT as helpful for at least some medical conditions. Although access and cost were common barriers, one quarter indicated that CBT required excessive time or effort. Most did not perceive CBT as stigmatizing and did not interpret provider recommendations for CBT as dismissive of their symptoms. Adults Participants with bowel DGBI reported greater perceived risk and familiarity with CBT than controls. However, receptiveness, interest, willingness, perceived stigma, and attitudes toward providers who recommended CBT did not differ across groups. Only individuals with other non‐DGBI GI diseases were more likely to have been offered CBT. In contrast, 90% of adults with bowel DGBI had never been offered CBT. After adjusting for psychosocial factors, adults with other non‐DGBI GI diseases were more likely to perceive CBT as helpful than controls, but receptiveness to CBT between those with bowel DGBI and controls did not differ and those with bowel DGBI expressed higher levels of willingness to try CBT.

Our findings are consistent with prior studies suggesting that a lack of widely available multidisciplinary resources is an important barrier to incorporating CBT into management of bowel DGBI. However, recent studies indicate that expansion may be possible. In one systematic review and network meta‐analysis [12], CBT in its multiple modalities (i.e., group sessions, telephone, self‐administered or minimal contact, face‐to‐face) was efficacious for treatment of IBS, and several studies have suggested that other resources including mobile applications or internet‐based CBT [25, 26] may be a reasonable alternative for expanding access to behavioral interventions more broadly for some. Nonetheless, although digital methods may increase availability of mental health services, they do not provide the personalized and monitored approach to BGBT as clinician‐guided BGBT, and further study is needed to confirm their long‐term efficacy [25, 27]. One unique aspect of this study is the observation that other aspects beyond access may hinder integration of CBT. Less than 10% of participants with bowel DGBI had ever been offered CBT in contrast to 27% of patients with other non‐DGBI GI diseases, despite the established efficacy of BGBT for improving symptoms [28] including pain [16] and quality of life [29] in patients with bowel DGBI. While reasons for low referral rates were not determined in this survey, it is possible that low referral rates may be representative of experiences of community‐based individuals rather than patients seeking care at specialty care sites. It is also possible that clinicians may be hesitant or uncomfortable in knowing how and when to refer patients to therapists and providers who are trained in BGBT and can guide personalized selection of appropriate behavioral therapy such as CBT for DGBI management. Indeed, a survey study among gastroenterology trainees found that only 31.6% of trainees were comfortable with knowing when to refer patients with DGBI to pyschogastroenterology [30]. Other possibilities such as concerns for perpetuating stigmatization or misconceptions regarding patients' attitudes toward CBT may also affect early incorporation of BGBT. Providing clinicians with strategies [10, 31] for open and empathetic discussions that frame CBT as an effective treatment is one initial step. Greater confidence in utilizing these approaches may also be achieved through an improved understanding of patients' concerns as one of our key findings was that patients with bowel DGBI reported a higher rate of perceived risks. But while there is some apprehension toward CBT, patients with bowel DGBI were still as receptive toward CBT as patients with other non‐DGBI GI diseases and controls. Understanding patients' apprehensions will be important, since CBT efficacy may be influenced by baseline anxieties or fears. A previous study demonstrated that trait anxiety and anxiety sensitivity, but not the number of psychiatric comorbidities, were linked to CBT success in refractory IBS [15].

**FIGURE 3:**
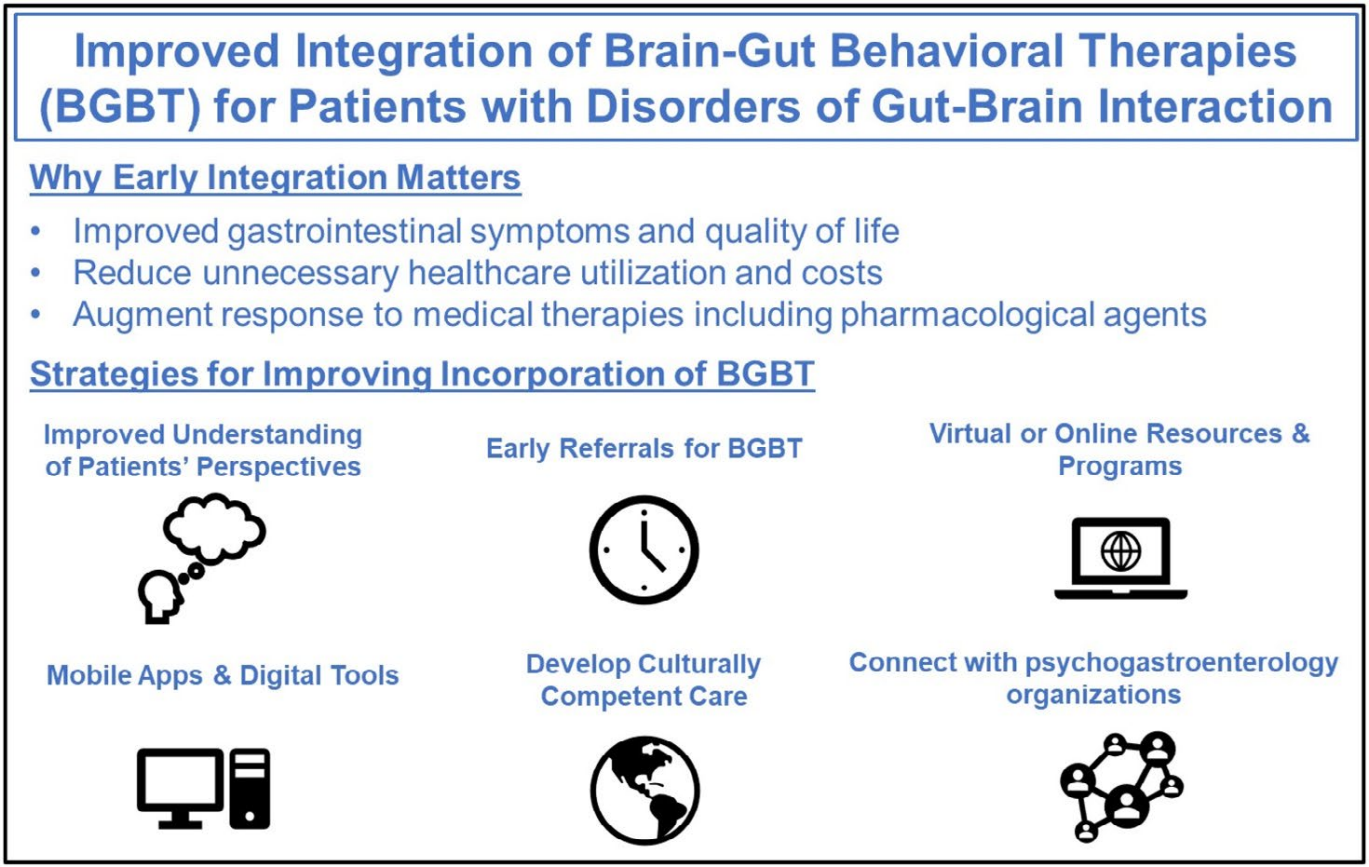
Proposed strategies for improving integration brain–gut behavior therapies among patients with disorders of gut–brain interaction.

Among patients with bowel DGBI, those with IBS expressed greater familiarity with CBT compared to other groups, while participants with FDr were more likely to believe that CBT could be helpful than those with IBS. IBS is characterized by abdominal pain, and it is possible that the perceived benefit of CBT lies in targeting hypervigilance to symptoms and illness‐specific cognitions that influence symptom severity rather than bowel dysfunction [10]. IBS is also among the most widely researched bowel DGBI and the role of BGBT is supported by society guidelines [32], which may also explain why patients with IBS are more frequently offered CBT than those with FC or FDr. Interestingly, patients with FDr were more likely to report that CBT could be helpful than those with IBS and less likely to cite access as a barrier. While reasons for these differences were not assessed, one possibility is that patients with FDr may associate symptoms with heightened stress and these relationships may be easier to discern given the singular symptom domain (diarrhea) that defines FDr [33]. Furthermore, despite the consensus regarding the benefit and cost effectiveness of CBT in IBS [34], it is often reserved for refractory cases [11, 35], which may reduce its relative impact or perceived utility. Our observations highlight the need for early referrals and scalable psychological care [36] to incorporate BGBT earlier in DGBI management rather than reserving it as a last resort (Figure 3).

Examination of psychological factors and sociodemographics further revealed participants identifying as non‐White and non‐Black racial minorities and those with higher levels of depression reported a lower perceived benefit with CBT, while Black participants expressed an increased willingness to consider CBT. The influence of race/ethnicity on attitudes toward BGBT in bowel DGBI has not previously been studied. However, recent work has shed light on potential differences by race for healthcare utilization in IBS [37] and others [29] have demonstrated that completion of intake visits in patients with chronic GI symptoms referred for psychological interventions did not differ by disease, age, or gender. Poorer outcomes in the score for work and social adjustment after CBT in men compared to women [38] and lower response to CBT in those with higher depressive symptoms [39] have also been reported. Further research will be necessary to develop culturally sensitive approaches to integrating BGBT in bowel DGBI care.

Study strengths include the use of validated questionnaires to identify patients with bowel DGBI by Rome IV criteria, a large and diverse sample, use of a quality control question, consideration of key confounders, and comparisons of different subtypes of bowel DGBI. Our study cohort consisted of a community‐based population rather than patients from a tertiary‐care site or specialty GI clinic where referrals to GI psychology and/or access to integrated care models may be greater. Hence, findings may be more generalizable to primary care or community‐based practices that frequently serve as the front‐line providers for patients with DGBI. Amazon's mTurk is a platform commonly used for social science survey administration and participants recruited through mTurk are representative of the general demographics of American adult internet users [19]. We further selected participants with high approval ratings, which are associated with enhanced attention and reliability of data [40].

There are limitations. We did not assess whether patient‐reported beliefs translated to actual behaviors or benefits. Knowledge or understanding of CBT was not assessed. There is potential for unmeasured confounding and selection bias. The use of convenience sampling and online recruitment may limit generalizability and without formal power calculations we cannot rule out that some of our between‐group comparisons may have been underpowered. However, the primary aim was descriptive characterization of perspectives toward CBT rather than formal hypothesis testing. Our findings may be used to inform future hypothesis‐driven research such as specific factors that explain patients' perspectives toward CBT or other BGBT and predictors of response. No demographic quotas were applied, possibly skewing representation of certain groups. However, mTurk respondents do not differ fundamentally from population‐based respondents after covariate adjustment as was done in our primary analyses [41]. Importantly, the distribution of these features allowed us to examine differences across sociodemographic groups. Self‐reported data may introduce recall bias and social desirability bias. However, participants were blinded to study objectives to minimize these effects. Bowel DGBI diagnoses were not confirmed by a physician diagnosis. However, we used validated diagnostic questionnaires, which have been utilized in prior epidemiological studies [3]. Among individuals with bowel DGBI, symptom severity was not assessed, which could influence perceptions. While most questionnaires were validated instruments, the questionnaire assessing CBT was created specifically for this study and not externally validated. However, our questionnaire was informed by literature review and expert consensus and included input from two GI psychologists to capture initial insights in an area where little prior research exists. Finally, this survey did not address BGBT or referrals for psychological/psychiatric care more broadly, and perceptions of gut‐specific vs. general psychotherapy may differ. Nevertheless, this distinction is unlikely to impact group comparisons as the focus on CBT was consistently emphasized for all.

In conclusion, many adults with bowel DGBI perceive CBT as potentially beneficial, yet barriers include apprehension, underutilization by providers, and limited availability of time and resources. Sociocultural factors may also influence patients' perspectives of CBT. Future studies should explore differences between bowel DGBI with and without pain‐predominant features, patients' perspectives and experiences with other forms of BGBT beyond CBT, as well as patient‐specific features or characteristics that may predict responses to various types of BGBT. These insights could assist providers in offering individualized and integrated care plans including CBT and other BGBT for patients. Further work should also explore how patient‐centered education, culture, language, and social factors shape perceptions of CBT.

## Author Contributions

Developing study concept: A.S. Planning study design: A.M.M., S.J., H.X., A.G. Data collection and study procedures: J.K., A.G. Data management: M.R.W., J.V., J.K., H.X., A.S. Data analysis and interpretation: D.I.V., H.X., A.S. Drafting the manuscript: D.I.V., M.R.W., J.V., A.S. Critically revising the manuscript: D.I.V., A.S. A.S. accepts full responsibility for the current version of the editorial and its submission to the journal. All authors approved the final version of the article, including the authorship list.

## Funding

This work was supported by the Indiana Clinical and Translational Sciences Institute, U54TR002358.

## Disclosure

A.S. serves as a consultant for Ardelyx, has served on an Advisory Board for Gemelli Biotech and Salix Pharmaceuticals, has served as a consultant or advisor for ModernGut, Medis Labs Inc, and Peterson Health Technology Institute.

Grant support: Indiana University Health Values Fund for Research Award and the Indiana Clinical and Translational Sciences Institute.

## Ethics Statement

The study was approved by the Indiana University Institutional Review Board.

## Conflicts of Interest

The authors declare no conflicts of interest.

## Supporting information


**Data S1:** nmo70283‐sup‐0001‐Supinfo.docx.

## Data Availability

The data that support the findings of this study are available from the corresponding author upon reasonable request.

## References

[1] F. Mearin , B. E. Lacy , L. Chang , et al., “Bowel Disorders,” Gastroenterology 150 (2016): 1393–1407. 10.1053/j.gastro.2016.02.031 27144627

[2] N. Tornkvist , O. S. Palsson , J. Hreinsson , et al., “Global Prevalence, Characterization and Impact of Functional Bowel Disorders,” American Journal of Gastroenterology (2025), 10.14309/ajg.0000000000003782. 41020663

[3] O. S. Palsson , W. Whitehead , H. Tornblom , et al., “Prevalence of Rome IV Functional Bowel Disorders Among Adults in the United States, Canada, and the United Kingdom,” Gastroenterology 158 (2020): 1262–1273. 31917991 10.1053/j.gastro.2019.12.021

[4] K. Bradford , W. Shih , E. J. Videlock , et al., “Association Between Early Adverse Life Events and Irritable Bowel Syndrome,” Clinical Gastroenterology and Hepatology 10 (2012): 385–390. 22178460 10.1016/j.cgh.2011.12.018 PMC3311761

[5] H. M. Staudacher , A. Mikocka‐Walus , and A. C. Ford , “Common Mental Disorders in Irritable Bowel Syndrome: Pathophysiology, Management, and Considerations for Future Randomised Controlled Trials,” Lancet Gastroenterology & Hepatology 6 (2021): 401–410. 33587890 10.1016/S2468-1253(20)30363-0

[6] J. Hendrix , D. Ranginani , A. M. Montero , et al., “Early Adverse Life Events and Post‐Traumatic Stress Disorder in Patients With Constipation and Suspected Disordered Defecation,” Neurogastroenterology and Motility 34 (2022): e14195. 34121276 10.1111/nmo.14195 PMC8715864

[7] E. M. M. Quigley , J. Horn , M. Kissous‐Hunt , R. A. Crozier , and L. A. Harris , “Better Understanding and Recognition of the Disconnects, Experiences, and Needs of Patients With Irritable Bowel Syndrome With Constipation (BURDEN IBS‐C) Study: Results of an Online Questionnaire,” Advances in Therapy 35 (2018): 967–980. 29946799 10.1007/s12325-018-0733-x PMC11343793

[8] W. D. Chey , L. Keefer , K. Whelan , and P. R. Gibson , “Behavioral and Diet Therapies in Integrated Care for Patients With Irritable Bowel Syndrome,” Gastroenterology 160 (2021): 47–62. 33091411 10.1053/j.gastro.2020.06.099

[9] L. Keefer , O. S. Palsson , and J. E. Pandolfino , “Best Practice Update: Incorporating Psychogastroenterology Into Management of Digestive Disorders,” Gastroenterology 154 (2018): 1249–1257. 29410117 10.1053/j.gastro.2018.01.045

[10] L. Keefer , S. K. Ballou , D. A. Drossman , G. Ringstrom , S. Elsenbruch , and B. Ljótsson , “A Rome Working Team Report on Brain‐Gut Behavior Therapies for Disorders of Gut‐Brain Interaction,” Gastroenterology 162 (2022): 300–315. 34529986 10.1053/j.gastro.2021.09.015

[11] J. M. Lackner , J. Jaccard , L. Keefer , et al., “Improvement in Gastrointestinal Symptoms After Cognitive Behavior Therapy for Refractory Irritable Bowel Syndrome,” Gastroenterology 155 (2018): 47–57. 29702118 10.1053/j.gastro.2018.03.063 PMC6035059

[12] C. J. Black , E. R. Thakur , L. A. Houghton , E. M. M. Quigley , P. Moayyedi , and A. C. Ford , “Efficacy of Psychological Therapies for Irritable Bowel Syndrome: Systematic Review and Network Meta‐Analysis,” Gut 69 (2020): 1441–1451. 32276950 10.1136/gutjnl-2020-321191

[13] A. C. Ford , B. E. Lacy , L. A. Harris , E. M. M. Quigley , and P. Moayyedi , “Effect of Antidepressants and Psychological Therapies in Irritable Bowel Syndrome: An Updated Systematic Review and Meta‐Analysis,” American Journal of Gastroenterology 114 (2019): 21–39. 30177784 10.1038/s41395-018-0222-5

[14] Z. Wang , S. P. H. Whiteside , L. Sim , et al., “Comparative Effectiveness and Safety of Cognitive Behavioral Therapy and Pharmacotherapy for Childhood Anxiety Disorders: A Systematic Review and Meta‐Analysis,” JAMA Pediatrics 171 (2017): 1049–1056. 28859190 10.1001/jamapediatrics.2017.3036 PMC5710373

[15] J. M. Lackner , J. Jaccard , and Group IBSOSR , “Factors Associated With Efficacy of Cognitive Behavior Therapy vs Education for Patients With Irritable Bowel Syndrome,” Clinical Gastroenterology and Hepatology 17 (2019): 1500–1508. 30613000 10.1016/j.cgh.2018.10.033 PMC6486458

[16] V. C. Goodoory , M. Khasawneh , E. R. Thakur , et al., “Effect of Brain‐Gut Behavioral Treatments on Abdominal Pain in Irritable Bowel Syndrome: Systematic Review and Network Meta‐Analysis,” Gastroenterology 167 (2024): 934–943. 38777133 10.1053/j.gastro.2024.05.010

[17] A. E. Kazdin , “Addressing the Treatment Gap: A Key Challenge for Extending Evidence‐Based Psychosocial Interventions,” Behaviour Research and Therapy 88 (2017): 7–18. 28110678 10.1016/j.brat.2016.06.004

[18] M. Hearn , P. J. Whorwell , and D. H. Vasant , “Stigma and Irritable Bowel Syndrome: A Taboo Subject?,” Lancet Gastroenterology & Hepatology 5 (2020): 607–615. 31924568 10.1016/S2468-1253(19)30348-6

[19] K. Mortensen and T. L. Hughes , “Comparing Amazon's Mechanical Turk Platform to Conventional Data Collection Methods in the Health and Medical Research Literature,” Journal of General Internal Medicine 33 (2018): 533–538. 29302882 10.1007/s11606-017-4246-0 PMC5880761

[20] O. S. Palsson , W. E. Whitehead , M. A. van Tilburg , et al., “Development and Validation of the Rome IV Diagnostic Questionnaire for Adults,” Gastroenterology 150 (2016): 1481–1491.

[21] J. Ware, Jr. , M. Kosinski , and S. D. Keller , “A 12‐Item Short‐Form Health Survey: Construction of Scales and Preliminary Tests of Reliability and Validity,” Medical Care 34 (1996): 220–233. 8628042 10.1097/00005650-199603000-00003

[22] A. S. Zigmond and R. P. Snaith , “The Hospital Anxiety and Depression Scale,” Acta Psychiatrica Scandinavica 67 (1983): 361–370. 6880820 10.1111/j.1600-0447.1983.tb09716.x

[23] C. A. Blevins , F. W. Weathers , M. T. Davis , T. K. Witte , and J. L. Domino , “The Posttraumatic Stress Disorder Checklist for DSM‐5 (PCL‐5): Development and Initial Psychometric Evaluation,” Journal of Traumatic Stress 28 (2015): 489–498. 26606250 10.1002/jts.22059

[24] V. J. Felitti , R. F. Anda , D. Nordenberg , et al., “Relationship of Childhood Abuse and Household Dysfunction to Many of the Leading Causes of Death in Adults. The Adverse Childhood Experiences (ACE) Study,” American Journal of Preventive Medicine 14 (1998): 245–258. 9635069 10.1016/s0749-3797(98)00017-8

[25] D. M. Brenner , A. M. Ladewski , and S. W. Kinsinger , “Development and Current State of Digital Therapeutics for Irritable Bowel Syndrome,” Clinical Gastroenterology and Hepatology 22 (2024): 222–234. 37743035 10.1016/j.cgh.2023.09.013

[26] M. P. Pathipati , L. L. Scott , A. C. Griser , and K. Staller , “Real‐World Outcomes for a Digital Prescription Mobile Application for Adults With Irritable Bowel Syndrome,” Neurogastroenterology and Motility 36 (2024): e14811. 38689434 10.1111/nmo.14811

[27] S. S. Hasan , S. Ballou , L. Keefer , and D. H. Vasant , “Improving Access to Gut‐Directed Hypnotherapy for Irritable Bowel Syndrome in the Digital Therapeutics' Era: Are Mobile Applications a “Smart” Solution?,” Neurogastroenterology and Motility 35 (2023): e14554. 36847206 10.1111/nmo.14554

[28] O. Altayar , V. Sharma , L. J. Prokop , A. Sood , and M. H. Murad , “Psychological Therapies in Patients With Irritable Bowel Syndrome: A Systematic Review and Meta‐Analysis of Randomized Controlled Trials,” Gastroenterology Research and Practice 2015 (2015): 549308. 25802514 10.1155/2015/549308 PMC4329838

[29] S. W. Kinsinger , S. Ballou , and L. Keefer , “Snapshot of an Integrated Psychosocial Gastroenterology Service,” World Journal of Gastroenterology 21 (2015): 1893–1899. 25684957 10.3748/wjg.v21.i6.1893 PMC4323468

[30] Y. Luo , R. E. Dixon , B. J. Shah , and L. A. Keefer , “Gastroenterology Trainees' Attitudes and Knowledge Towards Patients With Disorders of Gut‐Brain Interaction,” Neurogastroenterology and Motility 34 (2022): e14410. 35608084 10.1111/nmo.14410

[31] J. H. Feingold and D. A. Drossman , “Deconstructing Stigma as a Barrier to Treating DGBI: Lessons for Clinicians,” Neurogastroenterology and Motility 33 (2021): e14080. 33484225 10.1111/nmo.14080 PMC8091160

[32] B. E. Lacy , M. Pimentel , D. M. Brenner , et al., “ACG Clinical Guideline: Management of Irritable Bowel Syndrome,” American Journal of Gastroenterology 116 (2021): 17–44. 33315591 10.14309/ajg.0000000000001036

[33] J. Tack , “Functional Diarrhea,” Gastroenterology Clinics of North America 41 (2012): 629–637. 22917168 10.1016/j.gtc.2012.06.007

[34] E. D. Shah , L. Chang , J. K. Salwen‐Deremer , et al., “Contrasting Clinician and Insurer Perspectives to Managing Irritable Bowel Syndrome: Multilevel Modeling Analysis,” American Journal of Gastroenterology 116 (2021): 748–757. 33982945 10.14309/ajg.0000000000000989

[35] H. A. Everitt , S. Landau , G. O'Reilly , et al., “Cognitive Behavioural Therapy for Irritable Bowel Syndrome: 24‐Month Follow‐Up of Participants in the ACTIB Randomised Trial,” Lancet Gastroenterology & Hepatology 4 (2019): 863–872. 31492643 10.1016/S2468-1253(19)30243-2 PMC7026694

[36] J. Feingold , H. B. Murray , and L. Keefer , “Recent Advances in Cognitive Behavioral Therapy for Digestive Disorders and the Role of Applied Positive Psychology Across the Spectrum of GI Care,” Journal of Clinical Gastroenterology 53 (2019): 477–485. 31169757 10.1097/MCG.0000000000001234

[37] C. Silvernale , B. Kuo , and K. Staller , “Racial Disparity in Healthcare Utilization Among Patients With Irritable Bowel Syndrome: Results From a Multicenter Cohort,” Neurogastroenterology and Motility 33 (2021): e14039. 33263195 10.1111/nmo.14039

[38] L. van Kessel , D. Teunissen , and T. Lagro‐Janssen , “Sex‐Gender Differences in the Effectiveness of Treatment of Irritable Bowel Syndrome: A Systematic Review,” International Journal of General Medicine 14 (2021): 867–884. 33758534 10.2147/IJGM.S291964 PMC7979326

[39] D. A. Drossman , B. B. Toner , W. E. Whitehead , et al., “Cognitive‐Behavioral Therapy Versus Education and Desipramine Versus Placebo for Moderate to Severe Functional Bowel Disorders,” Gastroenterology 125 (2003): 19–31. 12851867 10.1016/s0016-5085(03)00669-3

[40] E. Peer , J. Vosgerau , and A. Acquisti , “Reputation as a Sufficient Condition for Data Quality on Amazon Mechanical Turk,” Behavior Research Methods 46 (2014): 1023–1031. 24356996 10.3758/s13428-013-0434-y

[41] K. E. Levay , J. Freese , and J. N. Druckman , “The Demographic and Political Composition of Mechanical Turk Samples,” SAGE Open 6 (2016): 6.

