# Supplementary material for: Perceived Utility of Cognitive Behavioral Therapy in People With Bowel Disorders of Gut–Brain Interaction
Source: Neurogastroenterol Motil. 2026 Mar 8;38(3):e70283. doi: 10.1111/nmo.70283 (PMC12967772; doi:10.1111/nmo.70283)
Supplement: Supplementary file 1 — Data S1: nmo70283‐sup‐0001‐Supinfo.docx. [file NMO-38-e70283-s001.docx]

**Supporting Information**

**Basic Questionnaire**

1. Please provide your mTURK number. [numerical value]
2. Would you like to participate in this volunteer research study? [yes/no]
3. Are you 18 years of age or older? [yes/no]
4. Do you have a known prior diagnosis of Crohn's disease, ulcerative colitis, celiac disease, cancer of the internal organs, microscopic colitis (lymphocytic/collagenous)? [yes/no, if yes please specify]
5. What was your sex (at birth)? [female/male]
6. What is your age (years)? [numerical value]
7. What at is your race? [African/African American/Asian/Caucasian/Native American/Native Hawaiian/Pacific Islander/Multiple/Prefer not to say; if other please specify]
8. Are you Hispanic or Latino [yes/no]
9. Marital status [single/married/long-term partner/divorced/widowed]
10. What is your highest education level? [less than high school degree/high school degree or equivalent such as GED/some college but no degree/associate degree/bachelor degree/graduate degree]
11. Do you currently smoke or use tobacco? [yes/no]
12. Do you consume alcohol? [never/rarely/occasionally/regularly/frequently]
13. What is your height (inches)? [numerical value]
14. What is your weight (lbs)? [numerical value]
15. What is your annual household income? [Less than $20,000/$20,000 to $34,999/$35,000 to $49,999/$50,000 to $74,999/$75,000 to $99,999/$100,000 to $149,999/$150,000 to $199,999/$200,000 or more]

**Questionnaire on Useful ness of Cognitive Behavioral Therapy**

Cognitive behavioral therapy or CBT is a type of therapy that involves meeting with a trained therapist to talk about your feelings and thoughts. Different types of therapists can perform CBT including psychiatrists, psychologists, and social workers. During CBT, a therapist will ask you to perform certain activities that may help decrease your symptoms. Activities can include discussions or conversations, breathing techniques, self-calming or relaxation techniques, learning how to practice positive thinking, or other strategies that can help you identify and manage thoughts and behaviors that might affect your health. CBT may be helpful in improving both physical and emotional health. Many medical professionals and researchers believe that CBT can be a useful and important part of treating a wide variety of physical symptoms and in promoting a person’s overall well-being.

1. Given the above description of CBT, we would like to know about your opinion on CBT. Do you believe that CBT can be helpful in promoting health?

A. Yes, CBT can be helpful for almost any condition, disease, or symptom.

B. Sometimes, CBT is probably helpful for many conditions, diseases or symptoms.

C. Neutral, CBT is neither helpful nor unhelpful.

D. Rarely, CBT probably is not helpful for most conditions, disease, or symptoms.

E. No, CBT is never helpful for any condition, disease, or symptom.

1a. *If selecting B or D*: What conditions do you believe can benefit from CBT?

A. Mostly psychological conditions (e.g. anxiety, depression, mood)

B. Mostly psychological conditions and some medical (or physical) conditions

C. Many psychological conditions and many medical (or physical) conditions

D. Mostly medical (or physical) conditions and some psychological conditions

E. Mostly medical (or physical) conditions only

2. Please briefly explain why you answered the way you did. [free text response]

3. What other reasons do you think could prevent you or someone else from seeking CBT? (you may choose more than one)

A. It is too much work and effort

B. It is too costly

C. There are no available professionals who are trained in CBT in my area

D. It would to too time-consuming

E. There are no obvious reasons why a person could not seek CBT (I do not see any obstacles to receiving CBT).

4. Some people may believe there is risk to participating in CBT. Which of the following options would you say pose the same degree of risk as CBT? (you can choose more than one)

A. No major risk beyond what would be encountered in every day life.

B. Medications

C. Surgeries

D. Non-surgical interventions

E. Physical therapy

5. Has your doctor or healthcare provider ever recommended CBT for you? [yes/no]

4a. If yes, for what reason (free text)

6. Do you or have you personally know anyone who has participated from CBT? [yes/no]

3a. If yes, did they benefit [yes/no]

7. If your medical doctor or healthcare provider recommended CBT for you as a treatment for a health condition or symptom, how would you perceive your doctor or provider?

A. He/she is interested providing the best care possible for my needs.

B. He/she does not believe my symptoms are real.

C. I would not have positive or negative feelings towards my doctor or provider based on this suggestion.

8. If your medical doctor or healthcare provider recommended CBT for you as a treatment, how likely would you be to consider trying CBT.

A. I would be highly likely to consider CBT.

B. I would be somewhat likely to consider CBT.

C. The suggestion would have no impact on my chances of considering CBT

D. I would be less likely to consider CBT.

E. I would be highly unlikely to consider CBT.

9. Please select the number that comes after 8 and before 10.

A. 0

B. 5

C. 7

D. 9

E. 11

10. Do you think that participating in CBT would affect how others viewed you?

A. Participating in CBT would definitely cause others to view me positively.

B. Participating in CBT could cause others to view me positively.

C. Participating in CBT would not affect how others viewed me.

D. Participating in CBT could cause others to view me negatively

E. Participating in CBT would definitely cause others to view me negatively.

11. If your doctor or provider recommended CBT for you as a treatment, how would you like CBT to be explained to you?

A. A general discussion between me and my doctor or provider

B. A one-time visit with the therapist to learn about CBT

C. Written information about CBT such as a hand-out

D. I would prefer to research CBT on my own time and learn about it independently

E. Something else [free text response]

12. How familiar were you with the concept of CBT before this study? (5 point scale, [1 = not at all familiar, 2 = slightly familiar, 3 = somewhat familiar, 4 = moderately familiar, 5 = very familiar])
